# Hemorrhage within the tympanic membrane without perforation

**DOI:** 10.1186/s40463-018-0300-0

**Published:** 2018-11-06

**Authors:** Chang-Hee Kim, Jung Eun Shin

**Affiliations:** Department of Otorhinolaryngology-Head and Neck Surgery, Konkuk University School of Medicine, Konkuk University Medical Center, 120-1 Neungdong-ro (Hwayang-dong), Gwangjin-gu, Seoul, 143-729 Republic of Korea

**Keywords:** Tympanic membrane, Hemorrhage, Hemotympanum, Head trauma, Barotrauma, Epistaxis

## Abstract

**Background:**

Hemotympanum refers to both the presence of blood in the middle ear cavity and to ecchymosis of the tympanic membrane (TM), and a systematic study of intra-TM (iTM) hemorrhage without bleeding in the middle ear cavity has not been conducted. The goals of our study were to analyze the causes of iTM hemorrhage without TM perforation or bleeding in the middle ear cavity, and to demonstrate the clinical characteristics of the disease.

**Methods:**

This Case series study included five patients with iTM hemorrhage between August 2014 and August 2017. An iTM hemorrhage was diagnosed when otoendoscopic examination demonstrated minor bleeding behind the intact TM, a hemorrhage was observed between the TM annulus and the epidermal layer, and temporal bone computed tomography revealed thickening of the TM without soft tissue density within the tympanic cavity or temporal bone fracture. Initial symptoms, and serial findings of otoendoscopy and pure tone audiometry (PTA) were investigated.

**Results:**

iTM hemorrhage developed due to blunt head trauma in two patients, descent barotrauma during scuba diving in two patients, and spontaneous epistaxis in one patient. Otalgia and ear fullness were the most common symptoms, but PTA showed no or minimal conductive hearing loss in all patients.

**Conclusions:**

An iTM hemorrhage may develop after blunt head trauma, barotrauma due to scuba diving, or spontaneous epistaxis; otological symptoms included otalgia, tinnitus, and aural fullness. An iTM hemorrhage resolved spontaneously without specific treatment, usually within 1 month.

## Background

Hemotympanum refers to both the presence of blood in the middle ear cavity and to ecchymosis of the tympanic membrane (TM). A temporal bone fracture due to blunt head trauma, therapeutic nasal packing, epistaxis, blood disorders, anticoagulant therapy, barotrauma, and otitis media are common causes of hemotympanum [1–5]. Previous studies of hemotympanum have focused on hemorrhages within the middle ear cavity. To our knowledge, a systematic study of intra-TM (iTM) hemorrhage without bleeding in the middle ear cavity has not been conducted, even though there have been reports of two cases [6, 7]. Although the thickness of TM is only about 0.1 mm, the TM has capillaries between outer epidermal layer and inner mucous layer. So it is reasonable that hemorrhage within the TM may be resulted from various causes such as head trauma and barotrauma. The purpose of the present study was to analyze the causes of iTM hemorrhage and demonstrate the clinical course of the disease.

## Methods

**Fig. 1:**
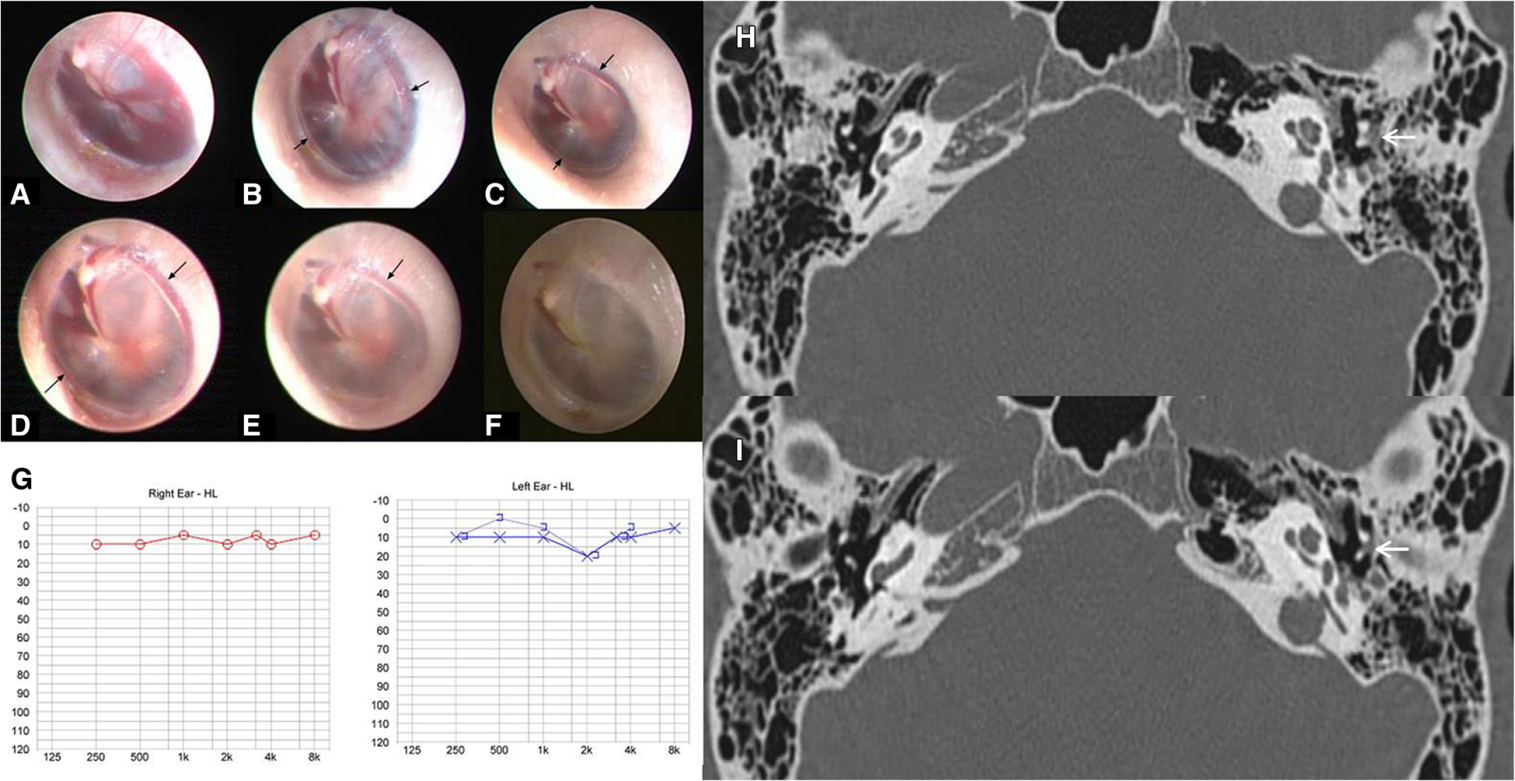
Serial otoendoscopic findings in patient 1. Note that minor bleeding is visible behind the intact left tympanic membrane (TM) and along the line between the annular ligament and epidermis of the TM (black arrows in panels (**a** - **e**). **a** Day 0, (**b**) Day 1, (**c**) Day 2, (**d**) Day 3, (**e**) Day 4, (**f**) Day 15. Pure tone audiometry tested the day of trauma (Day 0) revealed a minimum air-bone gap on the left side (**g**) which was improved after 1 month (**h**). The axial views of temporal bone computed tomography at Day 0 revealed a very small soft tissue density in the Prussak’s space (white arrows), consistent with iTM hemorrhage observed on otoendoscopic examination (**i** and **j**)

**Fig. 2:**
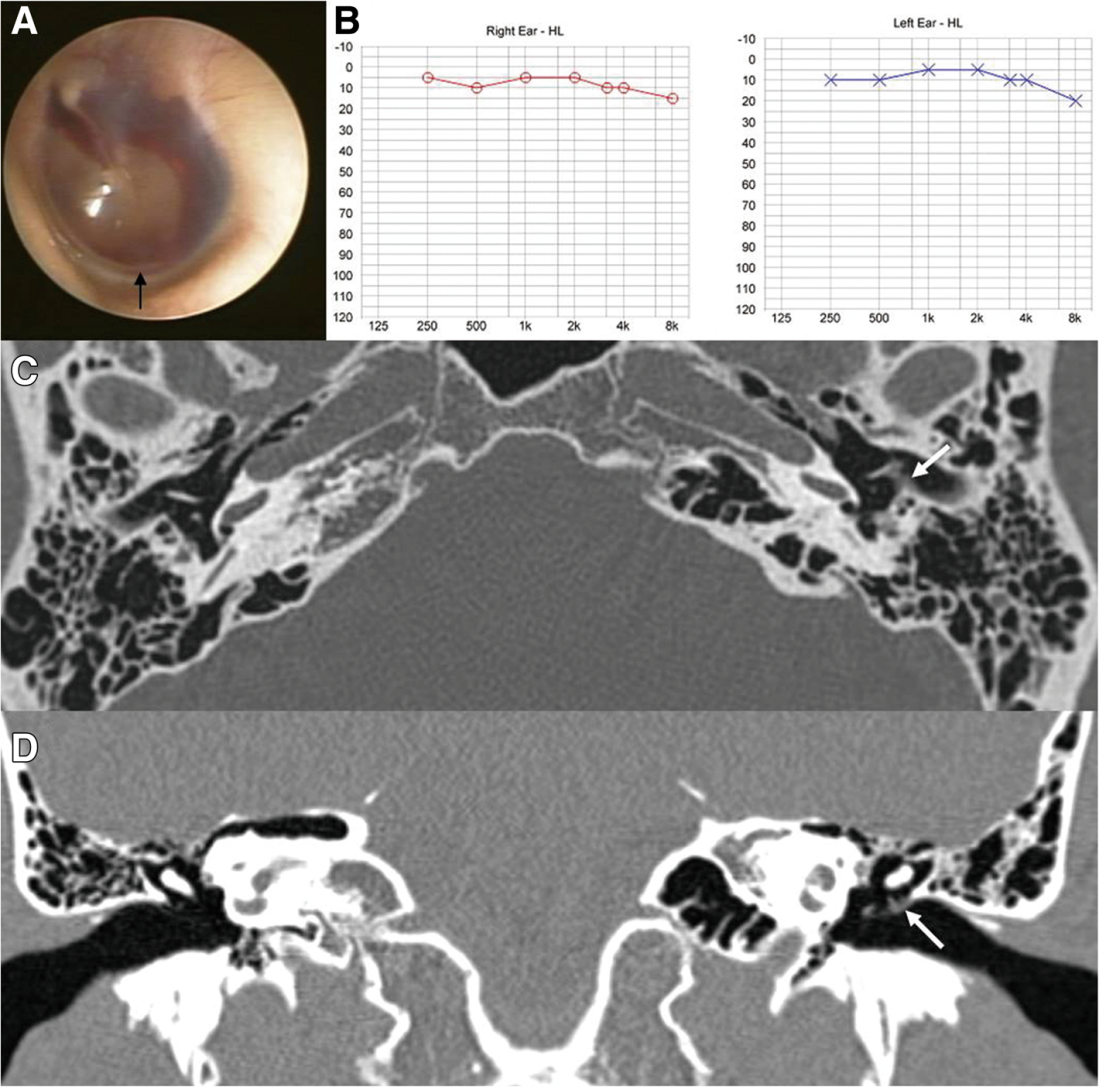
Otoendoscopic examination (**a**) of patient 2 revealed a small hemorrhage behind the intact left tympanic membrane (TM) and along the line between the tympanic annulus and epidermal layer of the TM (black arrow). Initial pure tone audiometry showed normal hearing on the left side (**b**), and follow-up pure tone audiometry after 1 week revealed no change (**c**). Temporal bone computed tomography demonstrated mild thickening of the left TM (white arrows) on axial (**d**) and coronal views (**e**), suggesting iTM hemorrhage

**Fig. 3:**
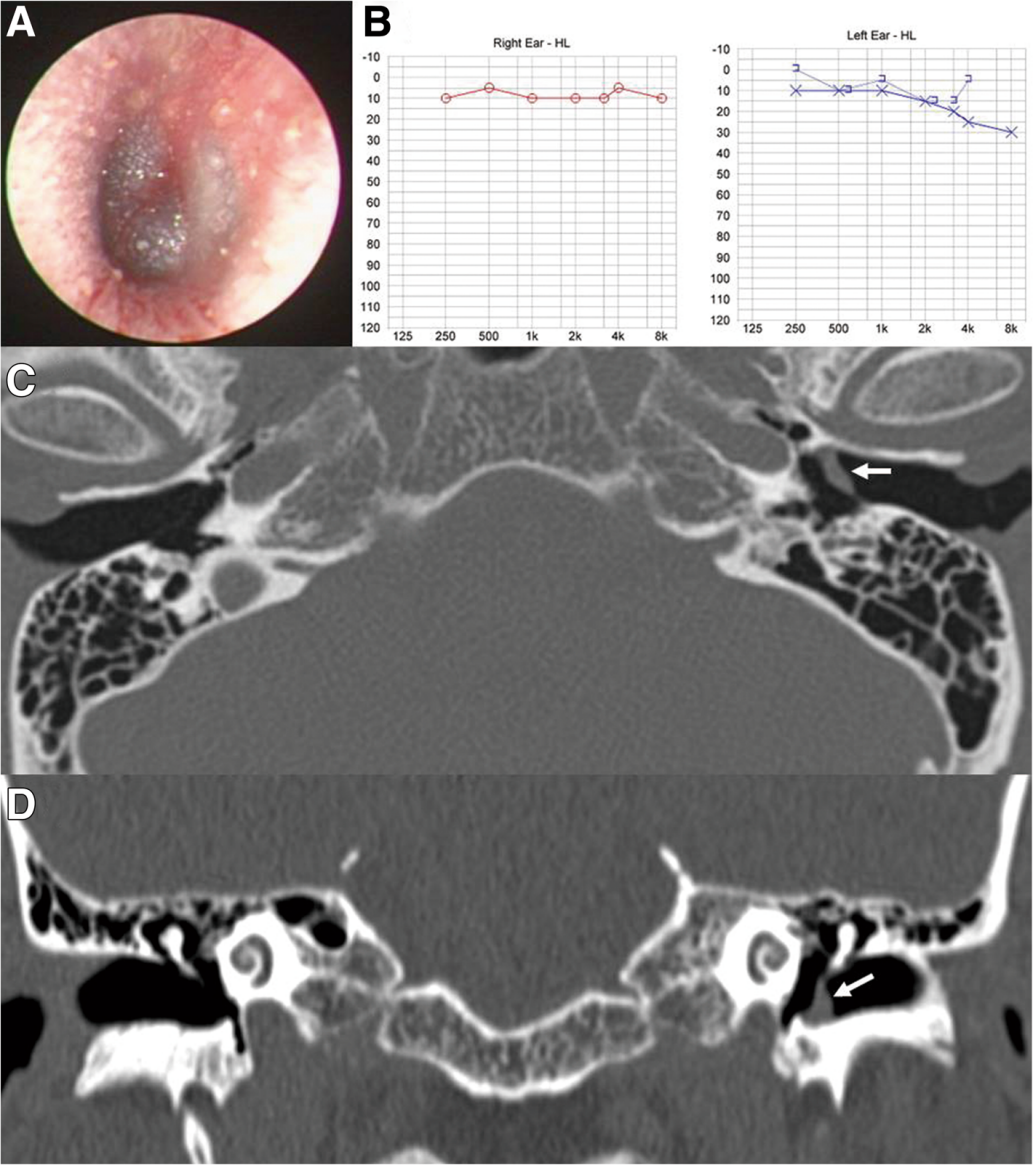
Otoendoscopic examination (**a**) of patient 3 showed bluish thickening of the anterior part of the intact left tympanic membrane (TM). Pure tone audiometry revealed mild conductive hearing loss on the left side (**b**), which was improved after 2 weeks (**c**). Temporal bone computed tomography revealed thickening of the left TM (white arrows) on axial (**d**) and coronal views (**e**), suggesting iTM hemorrhage

**Fig. 4:**
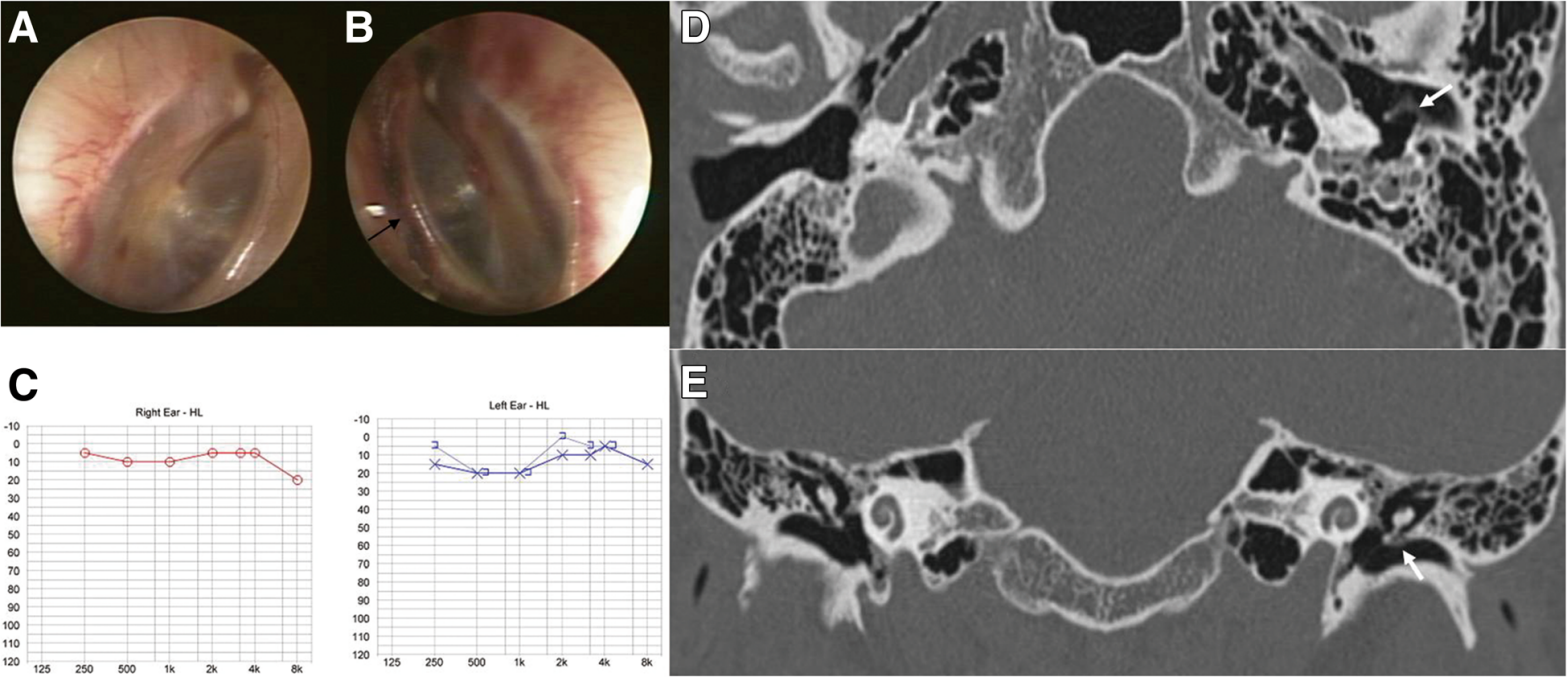
Otoendoscopic examination of patient 4 revealed minor bleeding along the manubrium of the malleus in the tympanic membrane (TM) on the right side (**a**), and a small hemorrhage behind the intact TM and along the line between the annular ligament and epidermis of the TM (black arrow, **b**). Pure tone audiometry revealed mild conductive hearing loss on the left side (**c**). Temporal bone computed tomography demonstrated mild thickening of the left TM on axial (**d**) and coronal views (**e**) (white arrows), suggesting iTM hemorrhage

**Fig. 5:**
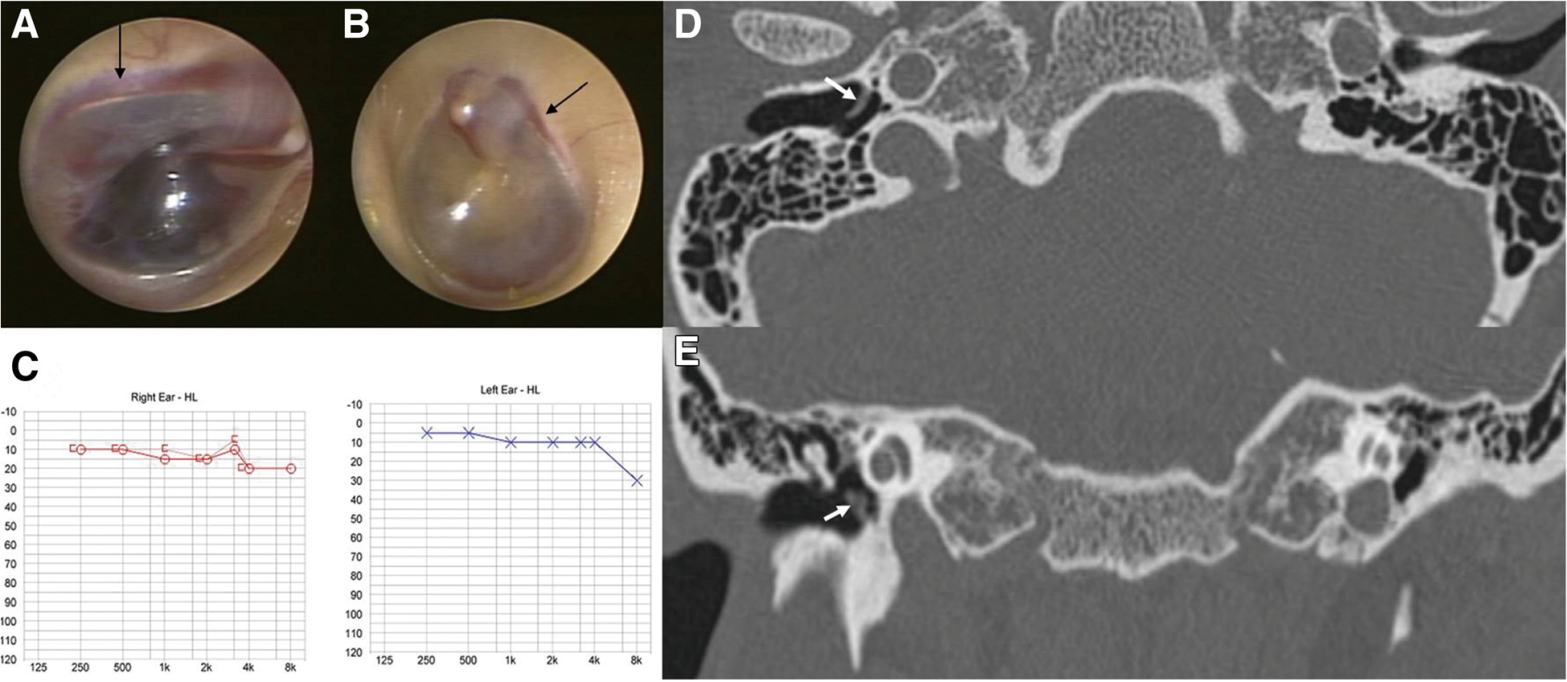
Otoendoscopic examination of patient 5 showed a small hemorrhage behind the intact tympanic membrane (TM) and along the line between the annular ligament and epidermis of the TM (black arrow) on the right side (**a**), and minor bleeding along the manubrium of the malleus and between the annular ligament and epidermis of the TM (black arrow) on the left side (**b**). Pure tone audiometry revealed no hearing loss on either side (**c**). Temporal bone computed tomography demonstrated mild thickening of the right TM on axial (**d**) and coronal views (**e**) (white arrows), suggesting iTM hemorrhage

We conducted a retrospective case series study of patients who showed iTM hemorrhage without perforation. Between August 2014 and August 2017, medical records of the patients who were diagnosed with hemotympanum or whose TM showed abnormal findings were retrospectively reviewed, and five patients of iTM hemorrhage without perforation were enrolled in this study. The presence of iTM hemorrhage was determined by otoendoscopy findings and temporal bone computed tomography (TBCT). On otoendoscopic examination, minor bleeding of a bright or dark red color was seen behind the intact TM; a hemorrhage was also observed between the tympanic annulus and the epidermis of the TM (Figs. 1, 2, 3, 4 and 5), as the tympanic annular ligament is embedded between the epidermal layer and the mucosal layer of the TM [8]. TBCT demonstrated thickening of the TM suggestive of an iTM hemorrhage without soft tissue density within the tympanic cavity, indicating a hemorrhage. Patients with TM perforation, middle ear effusion, or a hemorrhage in the tympanic cavity were excluded from the study. Patients who underwent intratympanic steroid injections were also excluded. Patients’ symptoms were reviewed, otoendoscopic and TBCT findings were evaluated, and audiometric results were serially compared in these patients with iTM hemorrhage. This study was approved by the Institutional Review Board (KUH1110068).

## Results

Clinical characteristics of five patients with iTM hemorrhage are summarized in Table 1. Among the five patients, iTM hemorrhage was associated with head trauma in two patients, barotrauma during scuba diving in two patients, and epistaxis in one patient. Otalgia and ear fullness were the most common symptoms, but pure tone audiometry (PTA) showed no or minimal conductive hearing loss in all patients. Follow-up duration was 1 week to 1 month in these patients.

**Table 1:** Clinical characteristics of the patients with intra-tympanic membrane hemorrhage

No.	Sex/Age	Side	Cause	Ear symptoms	Underlying diseases
1	M/19	Left	Head trauma (occipital area)	Left tinnitus, ear fullness, otalgia, vertigo	None
2	M/33	Left	Head trauma (left zygomaticomaxillary area)	Left otalgia, tinnitus, ear fullness	None
3	M/51	Left	Barotrauma (SCUBA diving)	Left severe otalgia, ear fullness	None
4	M/30	Both	Barotrauma (SCUBA diving)	Both otalgia, ear fullness	Warfarin medication due to previous aortic valve replacement surgery
5	F/35	Both	Epistaxis	Both mild ear fullness	None

Patient 1 (Table 1) was a previously healthy 19-year-old man who presented with otalgia, tinnitus, and ear fullness in the left ear associated with vertigo; symptoms developed after head trauma to the occipital area due to a fall from the horizontal bar. Otoendoscopic examination revealed a red hemorrhage behind the intact left TM and along the tympanic annulus between the annular ligament and epidermal layer of the TM (Fig. 1a-e), suggesting iTM hemorrhage. The right TM was normal. PTA demonstrated a minimum air-bone gap on the left side (Fig. 1g). TBCT was conducted at the day of first visit, and axial views of TBCT showed a very small soft tissue density in the Prussak’s space (Figures I, J) consistent with iTM hemorrhage observed on otoendoscopic examination without other abnormal findings. Neurologic examination revealed no focal neurologic deficit, but video nystagmography showed a peripheral, right-beating spontaneous nystagmus. The patient was hospitalized with a diagnosis of labyrinthine concussion and left iTM hemorrhage. He initially complained of severe vertigo accompanied by nausea and vomiting, but the symptoms improved greatly on the second day. A bithermal caloric test revealed 33% canal paresis on the left side. Over time, the ear fullness gradually resolved. The amount of hemorrhagic fluid in the TM decreased (Fig. 1a-f) and resolved within 1 month, and air conduction threshold was improved in PTA (Fig. 1h).

Patient 2 (Table 1) was a previously healthy 33-year-old man who visited our clinic with symptoms of left facial tenderness, otalgia, tinnitus, and a sensation of ear fullness in the left ear that had developed after left facial trauma 1 day previously. He did not complain of vertigo, and a neurologic examination revealed no abnormality. Dark red bleeding was observed through the intact TM on the left side on otoendoscopic examination (Fig. 2a). The thin line of a hemorrhage was seen between the tympanic annular ligament and epidermal layer, which was suggestive of an iTM hemorrhage. PTA showed normal hearing on both sides (Fig. 2b) despite symptoms of ear fullness and tinnitus. TBCT was conducted at the day of first visit, and it demonstrated mild thickening of the left TM on axial (Fig. 2d) and coronal views (Fig. 2e) without other abnormal findings, which was consistent with otoendoscopic findings indicating an iTM hemorrhage. One week later, the iTM hemorrhage had resolved without any complications and follow-up PTA showed no change (Fig. 2c).

Patient 3 (Table 1) was a previously healthy 51-year-old man who presented with ear fullness and severe otalgia of the left ear that developed during scuba diving 2 days previously. The diver had performed more than 30 dives. He felt that the descent during the most recent dive was faster than usual and experienced difficulty equalizing the pressure in his left ear with the surrounding water pressure. During descent, despite repeated middle ear autoinflation using the Valsalva maneuver, he experienced abrupt onset of severe otalgia on the left side. On otoendoscopic examination, bluish bulging of the anterior part of the left TM was observed, but TM perforation or bleeding was not noted (Fig. 3a). PTA revealed mild conductive hearing loss on the left side (Fig. 3b). TBCT was conducted at the day of first visit, which demonstrated thickening of the left TM on axial (Fig. 3d) and coronal views (Fig. 3e) without other abnormal findings, suggesting an iTM hemorrhage. Two weeks after the injury, the hematoma within the left TM resolved and the patient became asymptomatic, and PTA showed slight improvement (Fig. 3c).

Patient 4 (Table 1) had undergone aortic valve replacement due to aortic regurgitation 7 years previously and had been on anticoagulant medication since that procedure. He visited our clinic with a symptom of left ear fullness. He had gone scuba diving 10 days previously, and reported that he had felt otalgia and ear fullness on both sides during descent. Otoendoscopic examination showed minor bleeding along the manubrium of the malleus in the right TM (Fig. 4a) and minor hemorrhage behind the intact TM and along the annular ligament in the left ear (Fig. 4b). PTA revealed mild conductive hearing loss on the left side only (Fig. 4c). TBCT was conducted at the day of first visit, which revealed mild thickening of the left TM on axial (Fig. 4d) and coronal views (Fig. 4e) without other abnormal findings, suggesting an iTM hemorrhage. After 1 week, his ear discomfort resolved without treatment.

Patient 5 (Table 1) was a 35-year-old woman referred to our clinic with a complaint of epistaxis. Epistaxis began spontaneously on both sides without trauma while the patient was preparing breakfast. Though the bleeding was not massive, she immediately visited the emergency department. Her vital signs were stable and her past medical history was unremarkable. She was not on anticoagulant or NSAID medication, but took alprazolam occasionally when she suffered from sleep disturbance. A nasal endoscopic examination revealed mild bleeding in the Kiesselbach’s area of the bilateral nasal septa. Bleeding was easily controlled by electrocauterization, and antibiotic ointment was applied to the electrocauterized mucosa without nasal packing. After control of the epistaxis, the patient reported mild aural fullness in both ears, and otoendoscopic examination revealed a hemorrhage along the manubrium of the malleus and a small hemorrhage behind the intact TM and along the annular ligament of the right (Fig. 5a) and left TMs (Fig. 5b). The volume of the hemorrhage was greater in the right ear than in the left. PTA revealed normal hearing on both sides (Fig. 5c). TBCT was conducted at the day of first visit, which revealed mild thickening of the right TM on axial (Fig. 5d) and coronal views (Fig. 5e) without other abnormal findings, suggesting an iTM hemorrhage. After 10 days, the iTM hematoma was resolved, and the patient’s ear discomfort disappeared without treatment.

## Discussion

An iTM hemorrhage without TM perforation or bleeding in the tympanic cavity is so rarely observed that only five patients were enrolled in this study in 3 years. The causes of iTM hemorrhage included blunt head trauma in two patients, barotrauma due to scuba diving in two patients, and spontaneous epistaxis in one patient. Although all patients complained of ear fullness in the affected ear, PTA revealed no or minimal conductive hearing loss. Moreover, iTM hemorrhage spontaneously resolved without complication in all of the five patients.

The TM serves essential roles in sound transmission and middle ear protection. Its shape is irregularly round and slightly conical. The TM varies in thickness; it is thicker in the center and periphery than in the intermediate area, and thicker in the pars flaccida than in the pars tensa [9]. Three layers are clearly distinguished in both the pars tensa and pars flaccida: the outer epidermal layer, middle lamina propria, and inner mucous layer. The middle lamina propria is composed of outer radial fibers, inner circular fibers with parabolic fibers between the radial and circular fibers in the pars tensa, and loose connective tissue with abundant elastic and collagen fibers in the pars flaccida [9]. Although the structural organization of the middle lamina propria layer is distinct between the pars tensa and pars flaccida, the capillaries supplying the TM are located within the loose connective tissue of the middle lamina propria in both the pars tensa and pars flaccida [9–11].

Studies of the vascular distribution of the TM have been performed in humans [12–15] and other animals [16–19]. They described a dual arterial source supplying the TM; one from the tympanic annulus (peripheral ring plexus) and the other along the malleus handle (manubrial plexus). The vascular supply is derived from three major arteries: the anterior tympanic artery, deep auricular artery, and stylomastoid artery. The posterior half of the TM is more richly perfused than the anterior half. The outer epidermal layer of the TM is continuous with the epidermis of the external auditory canal skin, and the inner mucous layer is connected to the mucosa of the middle ear at the peripheral margin of the TM [8]. The TM annulus is a horseshoe-like, fibrocartilaginous structure that maintains attachment of the TM into the tympanic sulcus [8, 20]. As the TM annulus lies beneath the outer epidermal layer, slight bleeding between the TM annulus and the epidermal layer may indicate an iTM hemorrhage. In the present study, we determined the existence of iTM hemorrhage by otoendoscopic examination; minor bleeding of a bright or dark red color was seen behind the intact TM and between the TM annulus and the epidermal layer, in addition to TBCT findings that showed thickening of the TM with soft tissue density.

Most cases of hemotympanum caused by head trauma are also associated with temporal bone fracture [1]. In this case, bleeding within the tympanic cavity of the middle ear was observed on TBCT, and conductive or mixed hearing loss was revealed on audiometry. However, iTM hemorrhage without bleeding in the middle ear cavity caused by head trauma has not been reported. In the present study, two patients had iTM hemorrhage after head trauma; the region of trauma was the occipital area in one patient and the zygomaticomaxillary area in the other. It can be assumed that capillaries within the TM were injured due to head trauma.

Although scuba diving is popular as a recreational activity, it may expose participants to risks of injury, and more than 50% of all diving complications are associated with otologic pathology [21, 22]. Among them, middle ear barotrauma is the most common; it is associated with Eustachian tube dysfunction [23]. Two patients in this study who were experienced scuba divers developed iTM hemorrhage during descent despite their efforts at middle ear autoinflation. When the equilibration between middle ear and environmental pressures fails during descent, the pressure external to the TM exceeds that in the middle ear cavity and the TM bulges inward. An iTM hemorrhage may have developed due to injuries to the capillaries within the TM by acute retraction of the TM during descent.

Hemotympanum that occurred secondary to spontaneous epistaxis has been reported [2–4]. It developed after epistaxis without nasal packing in five patients. When posterior nasal packing was performed to control the epistaxis, Eustachian tube dysfunction due to peritubal lymphatic stasis was postulated as the cause [24]. However, in patients in previous studies, hemotympanum might have been caused by retrograde blood reflux via the Eustachian tube rather than by peritubal lymphatic stasis because nasal packing was not used [2–4]. The cause of iTM hemorrhage in our patient was unclear; we speculate that a sudden significant increase in blood pressure or attempting a Valsalva maneuver might have caused iTM hemorrhage in this patient.

The limitation of this study is that, owing to the nature of the case series study, there is no control group, intervention or outcome measured, and the authors suggested supposed causes of iTM hemorrhage without providing real pathophysiologic mechanisms.

## Conclusions

Although very rare, iTM hemorrhage may develop due to blunt head trauma, barotrauma, or spontaneous epistaxis. The patients with iTM hemorrhage complained of ear symptoms such as otalgia, tinnitus, and aural fullness. However, audiometry revealed no or very mild conductive hearing loss. An iTM hemorrhage resolved spontaneously without complications within 2 weeks in all patients.

## Abbreviations

iTM: Intra-tympanic membrane
PTA: Pure tone audiometry
TBCT: Temporal bone computed tomography
TM: Tympanic membrane
